# Expression of Immune System-Related Membrane Receptors CD40, RANK, BAFFR and LTβR is Associated with Clinical Outcome of Operated Non-Small-Cell Lung Cancer Patients

**DOI:** 10.3390/jcm8050741

**Published:** 2019-05-24

**Authors:** Foteinos-Ioannis D. Dimitrakopoulos, Anastasia E. Kottorou, Anna G. Antonacopoulou, Nikolaos Panagopoulos, Chrisoula Scopa, Melpomeni Kalofonou, Dimitrios Dougenis, Angelos Koutras, Thomas Makatsoris, Vassiliki Tzelepi, Haralabos P. Kalofonos

**Affiliations:** 1 Molecular Oncology Laboratory, Division of Oncology, Department of Medicine, Medical School, University of Patras, 26504 Patras, Greece; fodimitrak@yahoo.gr (F.-I.D.D.); agathonisiotisa@yahoo.gr (A.E.K.); antonac@gmail.com (A.G.A.); angkoutr@otenet.gr (A.K.); maktom@yahoo.com (T.M.); 2 Department of Cardiothoracic Surgery, Medical School, University of Patras, 26504 Patras, Greece; npanag72@gmail.com (N.P.); ddougen@med.uoa.gr (D.D.); 3 Department of Pathology, Medical School, University of Patras, 26504 Patras, Greece; cdscopa@med.upatras.gr (C.S.); btzelepi@upatras.gr (V.T.); 4 Institute of Biomedical Engineering, Imperial College London, London SW7 2AZ, UK; m.kalofonou@imperial.ac.uk

**Keywords:** CD40, BAFFR, RANK, LTβR, NSCLC, prognosis, survival, lung cancer, expression, protein, mRNA

## Abstract

An increasing number of studies implicates the NF-κB (Nuclear Factor of kappa light chain gene enhancer in B cells) alternative pathway in non-small-cell lung cancer (NSCLC). We assessed the clinical significance of CD40 (Tumor necrosis factor receptor superfamily member 5, TNFRSF5), BAFFR (B-cell activating factor receptor), RANK (Receptor activator of NF-κB) and LTβR (lymphotoxin β receptor) receptors, which activate the alternative pathway of NF-κB, in NSCLC. Evaluation of CD40, BAFFR, RANK and LTβR expression was performed based on the Cancer Genome Atlas (TCGA) and the Genotype-Tissue Expression (GTEx) datasets, while protein expression was assessed by immunohistochemistry in specimens from 119 operated NSCLC patients. *CD40* gene overexpression was correlated with improved five-year overall survival (OS) (*p* < 0.001), while increased *BAFFR* and *LTβR* mRNA levels were associated with worse OS in patients with adenocarcinomas (*p* < 0.001 and *p* < 0.001, respectively). Similarly, patients with adenocarcinomas exhibited a negative correlation between membranous BAFFR protein expression in carcinoma cells and three- and five-year survival (*p* = 0.021; HR, 4.977 and *p* = 0.030; HR, 3.358, respectively) as well as between BAFFR protein overexpression in cancer-associated fibroblasts (CAFs) and two-year survival (*p* = 0.036; HR, 1.983). Patients with increased LTβR nuclear protein staining or stage II patients with lower cytoplasmic LTβR protein expression had worse five-year OS (*p* = 0.039 and *p* = 0.008, respectively). Moreover, CD40 protein expression in tumor infiltrating lymphocytes (TILs) and CAFs was positively associated with metastatic spread while BAFFR protein expression in CAFs was negatively associated with bone metastasis (*p* = 0.041). Our data suggests that CD40, BAFFR, RANK and LTβR play an important role in NSCLC and further supports the role of NF-κB alternative pathway in NSCLC.

## 1. Introduction

The Nuclear Factor of kappa light chain gene enhancer in B cells (NF-κB) remains one of the most studied transcription factors in cancer biology due to its pivotal role in many cellular functions such as inflammation, metastasis, angiogenesis, metabolism, epithelial-mesenchymal transition and tumor cell survival [1]. In addition, NF-κB has been implicated not only in the development and progression of the disease, but also in treatment resistance [2,3]. Furthermore, the role of NF-κB in non-small-cell lung cancer (NSCLC) is well-documented and it has mainly been involved in proliferation, apoptosis, angiogenesis, inflammation and metastasis [4]. 

NF-κB signaling is mediated through NF-κB family members, mainly through the activation of two pathways, which are termed classical and alternative [5]. During the last decade, a continuously increasing number of publications from our and other groups have shed light on the role of the NF-κB alternative pathway in lung cancer. In particular, overexpression of the four main intracellular players of the alternative pathway (NF-κB2, RelB (transcription factor RelB), NIK (Mitogen-activated protein kinase kinase kinase 14) and Bcl3 (B-cell lymphoma 3 protein) has been identified in primary NSCLC compared to normal tissues and association of their expression with lymph node infiltration and overall survival (OS) has been revealed [6,7,8]. Additionally, we have previously reported that genetic variations of *NF-κB2* and *BCL3* are related to lung cancer risk and OS [6,9].

Although the NF-κB alternative pathway can be activated by viruses such as Epstein-Barr Virus (EBV) and the human T-cell leukemia-lymphoma virus (HTLV) [10], the principal activating signal comes from cell surface receptors, members of the tumor necrosis factor (TNF) family, such as CD40 (Tumor necrosis factor receptor superfamily member 5, TNFRSF5), B-cell activating factor receptor (BAFFR, TNFRSF13C), Receptor Activator of NF-κB (RANK, TNFRSF11A) and lymphotoxin β receptor (LTβR, TNFRSF3) [10]. These receptors have been implicated in major functions of the cancer cell as well as in the regulation of the immune system and anticancer immune reactions, with their roles having been studied more extensively in hematological than epithelial malignancies [11,12,13,14]. For instance, CD40 plays an important role in innate and adaptive immunity against cancer [11]. In addition, the BAFFR/BAFF axis has been associated with cancer progression, apoptosis and inflammation as well as with cancer cachexia [12]. Interestingly, RANK has been correlated with bone metastasis, sensitivity to chemotherapy, angiogenesis and modulation of immune response [13]. Moreover, signaling through LTβR has been correlated to formation of tertiary lymphoid organs, cytokine expression and cell proliferation, while it has also been associated with inflammation-related carcinogenesis [14]. Although the activation of these receptors has been associated mainly with the alternative pathway of NF-κB, they have also been implicated in the activation of other significant signaling pathways [13,15,16,17].

The purpose of this study was to evaluate alterations in the expression (in protein and mRNA level) of the major surface receptors of NF-κB alternative pathway, namely CD40, BAFFR, RANK and LTβR, and to investigate their clinical significance and prognostic value in NSCLC patients.

## 2. Materials and Methods

### 2.1. Study Design, Population, Tissue Specimens and Data Collection

Patients with NSCLC that had undergone curative resection of a lung tumor in the University Hospital of Patras between 2005 and 2010 were serially and retrospectively selected from the electronic database of the Department of Pathology of the University Hospital of Patras. Patients enrolled in this study were in a good performance status (PS = 0) and no data was available regarding driver mutations status. Patients who relapsed during the period of observation were mainly managed with chemotherapy. Ethical guidelines of the Helsinki Declaration (2013) were followed for the designation of this study [18]. The current study has been approved by the Scientific Committee and the Committee on Research and Ethics of the University Hospital of Patras (Greece, 22/18.2.2015). Hematoxylin and eosin (H&E) stained slides from the tumor were reviewed and tumor grade and histological stage were determined [19]. Formalin-fixed paraffin-embedded (FFPE) tissue specimens of the patients as well as adjacent non-neoplastic lung parenchyma were retrieved from the Pathology Department’s tissue archive.

The clinical data, including medical history, relapse and OS were retrieved from the archive of the Division of Oncology of the University Hospital of Patras. When data were missing from the clinical files, the patients were personally contacted. OS outcome was defined after an observation period of 60 months. 

### 2.2. Transcriptome Analyses of Public Datasets

To assess the gene expression changes of *CD40*, *BAFFR*, *RANK* and *LTβR*, data were analyzed from 483 lung cancer adenocarcinomas, 486 squamous-cell carcinomas cases and 347 normal cases provided by the Cancer Genome Atlas (TCGA) and the Genotype-Tissue Expression (GTEx) projects. The dot Box Plots were generated to compare the *CD40*, *BAFFR*, *RANK* and *LTβR* mRNA expression levels between the tumor and normal tissues of NSCLC patients. The Gene Expression Profiling Interactive Analysis (GEPIA) interactive tool was used for TCGA and GTEx data processing and visualization in this study [20]. Kaplan Meier (KM)-plotter was used for OS analysis and Kaplan-Meier curves based on the publicly available data on the expression values of *CD40*, *BAFFR*, *RANK* and *LTβR* genes from stages I-III NSCLC patients (Affymetrix probe IDs: 215346_at, 1552892_at, 207037_at and 203005_at, respectively). 

### 2.3. Immunohistochemical Analysis

The expression of CD40, BAFFR, RANK and LTβR was assessed by immunohistochemistry (IHC), as previously described [21]. Conditions and product details for each primary antibody are presented in Table 1. The Envision detection kit (DAKO, Glostrup, Denmark) and diaminobenzidine (DAB) chromogen (DAKO) were used for detection and visualization, respectively, according to the manufacturer’s instructions. Counterstaining of the sections was done by using dehydrated Harris’ hematoxylin (Merck, Kenilworth, NJ, USA). Protein blocking solution was used instead of the primary antibodies in consecutive sections to ensure specificity of the antibodies. The validation of immunohistochemical staining was performed using FFPE tissue specimens from lymphoma (CD40), normal liver (RANK) and normal lymph node (LTβR and BAFFR) as positive controls according to the manufacturer’s instructions.

### 2.4. Evaluation of Immunohistochemistry

Each slide was scored blindly by an experienced pathologist (VT). Evaluation of the immunohistochemical staining was performed as described previously [21]. Histological type and tumor grade were performed according to the 2015 WHO classification of lung tumors [22]. Representative areas were selected at low (×100) magnification. Cell counts were performed at a 400× magnification. At least 1,000 cells were counted in each section. Both epithelial and cancer-associated fibroblasts (CAFs) were evaluated. Cytoplasmic and membranous staining were separately evaluated for each marker on epithelial cells. Nuclear staining was also evaluated for LTβR. Cytoplasmic staining was evaluated for myofibroblasts. Evaluation was performed on a 0–100 scale with 10-point increments. The percentage (%) of cells showing positive staining was determined for each marker and each cellular department. 

The intensity of staining was also evaluated using a three-tiered scale. A total score was calculated by multiplying the percentage of positive cells by the intensity of staining (range 0–300). Other components of the tumor microenvironment (tumor-infiltrating lymphocytes (TILs), tumor-associated macrophages (TAMs), endothelial cells) were also evaluated and scored as positive or negative, based on the presence or absence of any staining. Microphotographs were obtained by Lumenera’s INFINITY HD digital camera (Lumenera Co, OTT, Canada) mounted on an Olympus BX41 microscope (Olympus Europa SE & Co., Hamburg, Germany). 

### 2.5. Statistical Analysis

Statistical analysis was performed by using the Statistical Package for Social Sciences version 17 (SPSS, Chicago, IL, USA). Categorical nominal variables were evaluated using the Chi-square test or Fisher exact test. The *t* test was used for continuous variables with normal distribution. Analysis for ordinal or continuous data was performed by using Kruskal-Wallis or the Mann-Whitney tests. Spearman’s correlation was used to assess associations between variables. The Kaplan-Meier curves and the log-rank test were used for plotting and comparison of survival rates, respectively. Multivariate analysis of the studied molecules was assessed by Cox regression analysis. The X-tile software was used in order to provide the best cut-off points [23]. Best cut-off points were used for all analyses, unless otherwise stated. *p* < 0.05 was considered statistically significant for all comparisons. 

## 3. Results

### 3.1. Patient Characteristics

Clinical and pathological characteristics of the cohort of 119 patients are presented in Table 2. The median age of patients was 66 years (range 42 to 84 years). Stages I to III were equally distributed. 67 cases had squamous cell carcinomas, 42 had adenocarcinomas and 10 had large cell carcinomas. Nodal metastatic status was known for 115 patients, of which 43.7% were found to have regional lymph nodes metastasis. Second, third and fifth year survival outcome were available in 117, 115 and 115 patients, respectively. Moreover, the regional relapse status in the two-year follow-up period was known for 30 patients, with 16 having relapsed.

### 3.2. Differences in CD40, BAFFR, RANK and LTβR Expression 

#### 3.2.1. *CD40*, *BAFFR*, *RANK* and *LTβR* Gene Expression was Similar in Different Groups of Patients and in Different Tissues

**Figure 1:**
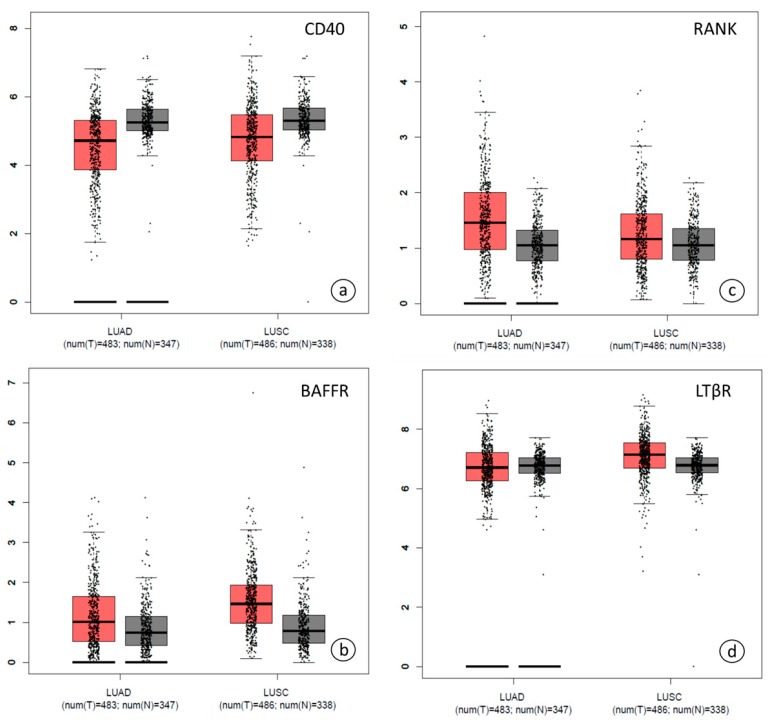
Dot Box plots in lung adenocarcinoma (LUAD) and lung squamous cell carcinoma (LUSC) subsets using TCGA and GTEx databases with regard to (**a**) *CD40*, (**b**) *BAFFR*, (**c**) *RANK* and (**d**) *LTβR* gene expression. Black lines at left lower parts represent zero point. Abbreviations: LUAD; lung adenocarcinoma, LUSC; lung squamous cell carcinoma, num; number, T; tumor, N; normal. Cancer tissues are depicted in red color, normal tissues in gray.

Gene expression data of *CD40*, *BAFFR*, *RANK* and *LTβR* provided by the TCGA and GTEx public databases were analyzed. [20]. Gene expression profiles of *CD40*, *BAFFR*, *RANK* and *LTβR* had similar patterns in the LUSC (lung squamous cell carcinoma) and LUAD (lung adenocarcinoma) subgroups analyzed and both were similar to control samples (Figure 1). 

#### 3.2.2. Subcellular Localization of CD40, RANK, BAFFR and LTβR

**Figure 2:**
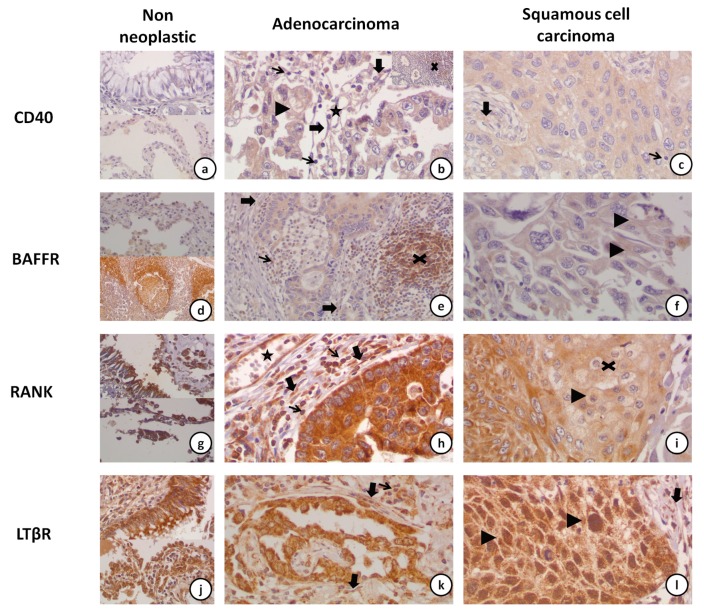
Representative immunohistochemical staining microphotographs of CD40, BAFFR, RANK and LTβR expression in NSCLC and adjacent, non-neoplastic tissue. (**a**) Rare CD40 positive cells in bronchial epithelium (upper part), pneumonocytes (lower part) and adjacent stroma (×40). (**b**) Moderate CD40 stain in an adenocarcinoma (×40). Stain is noted in the cytoplasm and occasionally the membrane (arrowhead) of the neoplastic cells. Tumor associated fibroblasts (thick arrow), and endothelial cells (star) are also positive. Tumor associated lymphocytes were usually negative (thin arrow). An intensely positive lymphoid aggregate (X) in the periphery of an adenocarcinoma is shown in the inset (×20). (**c**) Moderate CD40 stain in a squamous cell carcinoma (×40). Tumor associated fibroblasts (thick arrow) were positive, whereas tumor associated lymphocytes (thin arrow) were usually negative. (**d**) BAFFR is expressed in pneumonocytes (upper part). Intense expression is noted in marginal and mantle zone lymphocytes in a hilar lymph node (lower part) (×20). (**e**) Moderate BAFFR expression in the neoplastic cells of an adenocarcinoma, the tumor associated lymphocytes (thin arrow) and the tumor associated fibroblasts (thick arrow) (×20). An adjacent lymphoid aggregate is strongly positive (X). (**f**) Faint BAFFR expression in a squamous cell carcinoma (×40). Faint membranous expression is seen in some of the tumor cells (arrowhead). (**g**) Strong RANK expression in bronchial epithelium (upper part), pneumonocytes (lower part) and adjacent stroma (×40). (**h**) RANK is strongly expressed in the neoplastic cells, the tumor associated fibroblasts (thick arrow), the endothelial cells (star) and the tumor associated lymphocytes (thin arrow) in an adenocarcinoma case. (**i**) Faint to moderate RANK expression in a squamous cell carcinoma (×40). Note that the more differentiated cells in the center of the tumor nodule show less intense staining (X). Rare cells display membranous staining (arrowhead). (**j**) Strong LTβR expression in bronchial epithelium (upper part), pneumonocytes (lower part) and adjacent stroma (×40). (**k**) LTβR is strongly expressed in the neoplastic cells, the tumor associated fibroblasts (thick arrow) and the tumor associated lymphocytes (thin arrow) in an adenocarcinoma case. (**l**) Cytoplasmic and nuclear (arrowhead) expression of LTβR in the neoplastic cells of a squamous cell carcinoma case (×40). Expression is also seen in tumor associated fibroblasts (thick arrow).

Expression of CD40 was noted in the cytoplasm and the membrane of neoplastic cells and was faint (intensity 1+) in most of the cases. CAFs also showed faint cytoplasmic staining. Immunostaining was very rare in scattered lymphocytes within the tumor. However, lymphoid aggregates within the tumor or adjacent non neoplastic parenchyma were strongly positive. Positive staining was also found in the cytoplasm of vascular muscle and endothelial cells as well as in the cytoplasm of macrophages (Figure 2a–c). 

Regarding BAFFR expression, neoplastic cells had a finely dot-like cytoplasmic staining. Membranous staining was also noted in a few cases. CAFs showed cytoplasmic staining. BAFFR staining was intensely positive in lymphoid aggregates and mantle zone lymphocytes (in lymphoid follicles formed in adjacent tissue and in lymph nodes). In addition, staining in lymphocytes was detected in both the cytoplasm and membrane, while in macrophages staining was restricted to the cytoplasm. Furthermore, BAFFR was expressed in the cytoplasm of vascular muscle and in endothelial cells (Figure 2d–f).

Regarding RANK, the staining was mainly cytoplasmic with a coarse dot-like pattern in neoplastic cells and CAFs. In some cells, dots were lined close to the cell membrane. In lymphocytes, the localization of staining was cytoplasmic and membranous, while in macrophages staining was limited to the cytoplasm. Similarly to the other markers, RANK was expressed in vascular muscle and endothelial cells (Figure 2g–i).

LTβR signal had a coarse dot-like cytoplasmic staining in neoplastic cells. Membranous staining was seen in some cases. However, interestingly, nuclear staining was seen in most of the cases. Cytoplasmic and rare membranous immunostaining was detected in vascular muscle and endothelial cells. Lymphocytes, macrophages and CAFs showed cytoplasmic staining in all specimens (Figure 2j–l).

#### 3.2.3. CD40 and BAFFR Protein Expression Was Lower in NSCLC 

Immunohistochemical staining for CD40 in the cytoplasm and membrane of the tumor cells was noted in 99% and 49% of the cases, respectively (Figure 2b–c). Expression of CD40 in both compartments (cytoplasm and cell membrane), was significantly lower than that in non-neoplastic lung parenchyma (*p* = 0.005 and *p* < 0.001, respectively). 

BAFFR expression was detected in the cytoplasm of the tumor cells in all cases, while expression in the tumor cell membrane was noted only in 26.7% of cases (Figure 2e–f). Furthermore, lower membranous BAFFR expression was observed in tumor cells compared to tumor-adjacent parenchyma (*p* = 0.001), while the cytoplasmic staining was similar between tumor and adjacent tissues (*p* = 0.689).

Immunostaining for RANK in the cytoplasm and the membrane of tumor cells was positive in 100% and 64.1% of cases, respectively (Figure 2h–i). No difference in cytoplasmic and membranous RANK signal was found between cancerous and non-neoplastic tissues (*p* = 0.289 and *p* = 0.400, respectively).

Membranous, cytoplasmic and nuclear staining for LTβR was observed in 34.6%, in 100%, and in 86.5% of cases, respectively, and no difference was observed in the levels of expression between cancer cells and adjacent non-neoplastic cells (Figure 2j–l, *p* = 0.211, *p* = 0.602, *p* = 0.638, respectively).

#### 3.2.4. Correlation of CD40, BAFFR, RANK and LTβR Expression amongst the Cells of the Tumor Microenvironment (Tumor Cells and TILs/TAMs/CAFs)

CD40 staining was positive in 5, 96.6 and 97.5% of the cases in TILs, TAMs and CAFs, respectively. Cytoplasmic and membranous CD40 expression in carcinoma cells was positively associated with expression in CAFs (both *p* < 0.001). Moreover, CD40 staining in fibroblasts was positively correlated with its expression in TILs and macrophages (*p* = 0.006 and *p* = 0.011, respectively). Furthermore, cytoplasmic and membrane CD40 expression of carcinoma cells was positively correlated with each other (*p* = 0.017). 

TAMs and CAFs were positive for BAFFR in all samples, whereas TILs expressed BAFFR in 83% of the cases. BAFFR cytoplasmic expression in tumor cells was positively correlated with stromal BAFFR expression (*p* < 0.001). No other association regarding BAFFR expression in the cells of the tumor microenvironment was observed.

Regarding RANK, TILs, TAMs and CAFs, they were found positive for BAFFR in all cases. A negative association between cytoplasmic and membrane RANK expression in tumor cells (*p* < 0.001) was noted.

With regard to LTβR, all tissue specimens were positive for LTβR expression in CAFs, TILs and TAMs. Cytoplasmic LTβR expression was negatively correlated with membranous and nuclear expression in neoplastic cells (*p* < 0.001 and *p* = 0.001, respectively).

### 3.3. Association of CD40, BAFFR, RANK and LTβR Expression with Clinical Outcome

#### 3.3.1. *CD40*, *BAFFR* and *LTβR* mRNA Levels were Associated with OS

**Figure 3:**
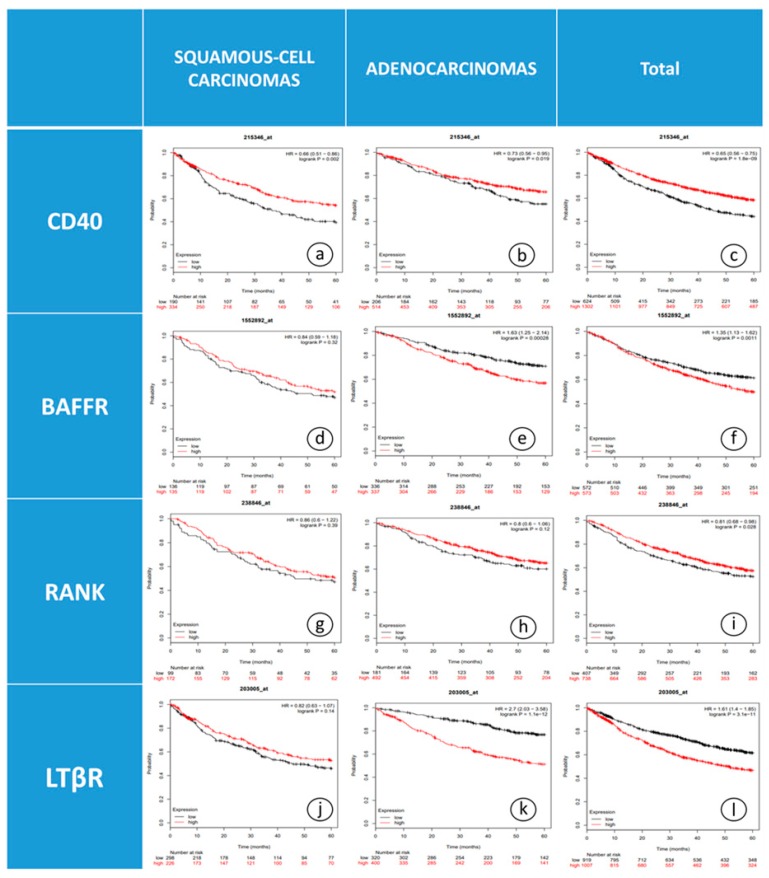
Kaplan-Meier curves depicting OS of NSCLC patients in relation to mRNA levels of: (**a**) *CD40* in SC patients, (**b**) *CD40* in ADC patients, (**c**) *CD40* in the whole cohort, (**d**) *BAFFR* in SC patients, (**e**) *BAFFR* in ADC patients, (**f**) *BAFFR* in the whole cohort, (**g**) *RANK* in SC patients, (**h**) *RANK* in ADC patients, (**i**) *RANK* in the whole cohort, (**j**) *LTβR* in SC patients, (**k**) *LTβR* in ADC patients and (**l**) *LTβR* in the whole cohort. Abbreviations: SC; squamous cell carcinoma, ADC; adenocarcinoma.

Survival analysis using KM-plotter for the JetSet probe set alone and best cut-off option for all available datasets combined, revealed that elevated CD40 gene expression was correlated with improved five-year OS of NSCLC patients (*n* = 1926) with squamous cell carcinomas and adenocarcinomas (Figure 3a–c, *p* = 0.002 and *p* = 0.019, respectively).

Evaluation of BAFFR expression in relation to OS, using the median as a cutoff in NSCLC patients (*n* = 1145), showed that BAFFR expression over the median was significantly associated with worse OS (Figure 3f, *p* = 0.001). Further analysis by histological subtype stratification revealed that this relation is limited to patients with adenocarcinomas (Figure 3e, *p* < 0.001) and not in the cases of squamous cell carcinomas (Figure 3d, *p* = 0.32). 

Additionally, a statistically significant positive association was observed between *RANK* mRNA expression and OS (*n* = 1145, Figure 3i, *p* = 0.026). On the contrary, upon further stratification with histology, no correlation was found between RANK gene expression and OS in patients with squamous-cell carcinomas or adenocarcinomas (Figure 3g–h, *p* = 0.95 and *p* = 0.12, respectively). 

With regard to *LTβR* expression, patients with adenocarcinomas and high *LTβR* expression had poor clinical outcome after a five-year observation period (Figure 3k, *p* < 0.001). On the contrary, OS was not affected by *LTβR* expression in patients with squamous cell carcinomas (*n* = 1926, Figure 3j, *p* = 0.42). 

#### 3.3.2. BAFFR, RANK and LTβR Protein Expression Were Associated with OS 

**Figure 4:**
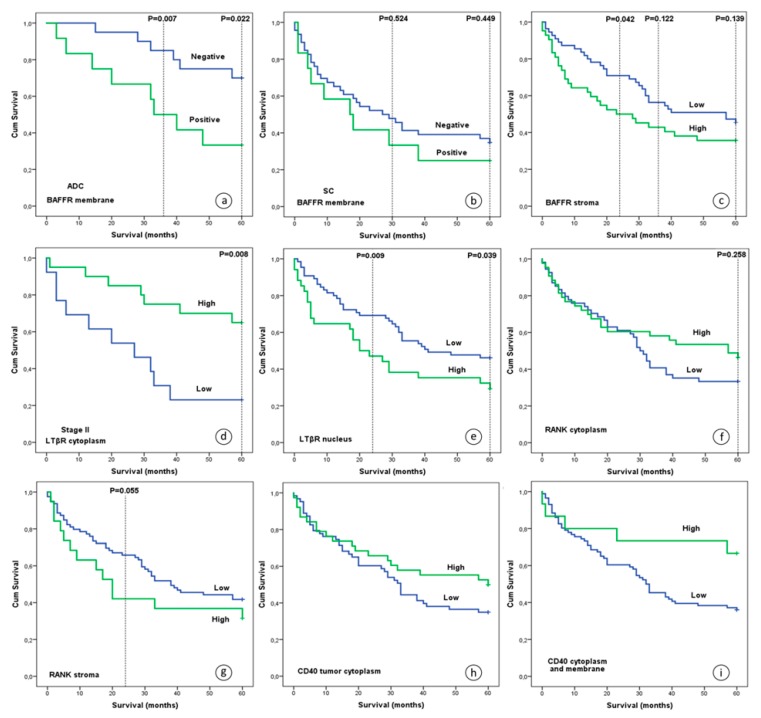
OS of NSCLC patients after five years of observation in relation to protein levels: (**a**) membranous BAFFR in adenocarcinomas, (**b**) membranous BAFFR in squamous cell carcinomas, (**c**) BAFFR in stroma cells, (**d**) LTβR in the cytoplasm of tumor cells of stage II patients, (**e**) nuclear LTβR in tumor cells, (**f**) cytoplasmic RANK in tumor cells, (**g**) RANK in CAFs, (**h**) CD40 in the cytoplasm of tumor cells and (**i**) cytoplasmic and membranous CD40 in tumor cells. Abbreviations: ADC; adenocarcinoma, SC; squamous cell carcinoma.

With regard to membranous BAFFR, patients with adenocarcinomas and no BAFFR expression had better three- and five-year OS compared to patients whose tumors expressed BAFFR (Figure 4a, *p* = 0.007 and *p* = 0.022, respectively). Furthermore, multivariate analysis with Cox regression models and age, gender and primary location as coefficients, further supported the previous findings (*p* = 0.021; HR, 0.201; 95% CI, 0.051-0.785 and *p* = 0.030; HR, 0.298; 95% CI, 0.100–0.887, respectively). No similar correlation was observed for three- and five-year survival outcome in patients with squamous cell carcinomas (Figure 4b, *p* = 0.524 and *p* = 0.449, respectively).

On the other hand, increased BAFFR expression in CAFs was correlated with worse two-year survival (Figure 4c, *p* = 0.042), but not with three- and five-year OS (*p* = 0.122 and *p* = 0.139, respectively). This finding persisted after multivariate analysis with age, gender, stage and primary location as coefficients (*p* = 0.036; HR, 1.983; 95% CI, 1.045–3.763).

In addition, univariate analysis revealed that lower cytoplasmic expression levels of LTβR in tumor cells of stage II patients were associated with decreased five-year OS (Figure 4d, *p* = 0.008). This finding remained significant after performing a Cox regression analysis (*p* = 0.008; HR, 4.004; 95% CI, 1.438–11.151). In addition, LTβR nuclear staining was associated with five-year OS (Figure 4e, *p* = 0.039), with increased expression being correlated with worse survival. 

No statistically significant association was found between RANK expression in the cancer cell cytoplasm and five-year survival, although the separation of the two curves in the Kaplan Meier plot after the second year of observation suggests a possibly better five-year OS for patients with higher expression after an initial two-year period (Figure 4f, *p* = 0.258). Interestingly, increased expression of RANK in the CAFs was marginally associated with poor two-, but not three- and five-year survival outcome (Figure 4g, *p* = 0.055, *p* = 0.186 and *p* = 0.254, respectively).

CD40 expression in the cytoplasm or on the membrane was not significantly associated with five-year OS, although a trend was noted for a relation between high CD40 levels and improved five-year OS, compared to patients with lower expression rates (Figure 4h–i, *p* = 0.092 and *p* = 0.059, respectively).

#### 3.3.3. CD40 and BAFFR Protein Expression Correlated with Development of Metastasis

CD40 and BAFFR protein expression were correlated with metastasis development during a two-year follow up. In particular, CD40 immunodetection in TILs was positively associated with metastatic spread in adrenal glands (*p* = 0.003), liver (*p* < 0.001) and brain (*p* = 0.048). Furthermore, CD40 expression in CAFs was also positively related to liver metastasis (*p* = 0.018). In addition, higher BAFFR expression in CAFs was associated with lower rate of bone metastasis (*p* = 0.041), while no correlation was found between BAFFR expression in tumor cells, TILs, TAMs and distal or nodal metastatic spread.

### 3.4. Correlations between Protein Expression and Clinicopathological Characteristics

Patients with higher pathological disease stage had lower expression of CD40 in neoplastic cells (*p* = 0.040). Expression of CD40 and LTβR in CAFs was also correlated with disease stage (*p* = 0.029, *r*= −0.222 and *p* = 0.045, *r*= 0.193, respectively). In addition, squamous cell carcinomas had lower expression levels of membranous RANK and higher levels of nuclear LTβR compared to adenocarcinomas and large cell carcinomas (*p* = 0.018 and *p* = 0.020, respectively). Membranous RANK and nuclear LTβR expression also correlated with gender. Female patients displayed increased RANK and decreased LTβR expression compared to male patients (*p* = 0.011 and *p* = 0.014, respectively). Moreover, RANK expression was lower in the centrally located better differentiated cells compared to the peripherally located less differentiated ones, in the tumor nodules of squamous cell carcinoma. No association was detected between CD40, BAFFR, RANK and LTβR expression and other clinicopathological characteristics.

## 4. Discussion

Lung cancer remains the leading cause of cancer-related deaths worldwide. Although the study of the underlying molecular mechanisms has offered opportunities for new treatment approaches, the molecular pathogenesis of the disease remains poorly understood. Research interest during the last decade has focused on the role of the NF-κB alternative pathway in cancer initiation, progression and treatment resistance. In this context, we investigated the expression profiles of CD40, BAFFR, RANK and LTβR in NSCLC tissue specimens and their significance on the clinical outcome. 

One of the major findings of our study is the deregulation of the CD40 and BAFFR protein expression in NSCLC. Findings on CD40 are consistent with the results published by Keidai et al., in which 51.9% of NSCLC tissue specimens [24] and 78% of lung cancer cell lines (14 of 18) expressed CD40 [25]. Furthermore, the expression of CD40 has been documented in many other carcinomas, such as melanomas [26], hepatocellular carcinomas [27] thyroid [28], bladder [29], colon [30], esophageal squamous cell [31], ovarian [32], gastric [33], cervical [34], breast and pancreatic cancer [35], as well as in the vast majority of hematological malignancies [36,37].

With respect to BAFFR, there has been limited research regarding B-cell physiology and hematological malignancies [38,39,40] and little is known about the association with epithelial cancers. Although Pelekanou et al. have not seen evidence of a positive expression of BAFFR in a variety of human cancers (breast [41], renal cell [42], gliomas [43]), BAFFR mRNA has been detected in the same cancers as revealed by TCGA data analysis as well as in lung cancer specimens in the current study. Furthermore, BAFFR expression has been detected in osteosarcomas [44]. In addition, an increased BAFFR expression in lung cancer may be inferred based on the report from Koizumi et al. that the expression of BAFF, the most important ligand of BAFFR, is associated in TILs with the expression of BAFFR [45] and that BAFF has remarkably higher expression in T lymphocytes from lung cancer-associated pleural effusions [46].

Cytoplasmic RANK detection was observed in all samples, while membranous signal was observed in a subset of the tumors. Our findings with regard to RANK are in agreement with published data, where membranous RANK expression was noted in 59.6% of 52 NSCLC cases [47]. RANK expression in lung cancer has also been reported by other research groups as well as in other cancer types [13]. RANK expression in tumor cells has been associated with cell proliferation, EMT and cell migration while it functions as cell chemoattractant [13]. In addition, its expression has been reported to induce chemoresistance [48]. However, no significant difference was observed in RANK levels between tumor and adjacent non neoplastic tissue in our cohort. As we mentioned above, RANK was also detected in TAMs as well as in TILs. RANK expression in TAMs has also been described in breast adenocarcinomas where RANK was strongly expressed [49]. 

With regard to LTβR, cytoplasmic expression was noted in CAFs, TILs and TAMs in all tissue samples while nuclear staining was observed in cancer cells in a very high percentage of cases (86.5%). Our findings are in agreement with published reports in other types of cancer. In particular, LTβR expression has been noted in ovarian cancer cells and their CAFs [50] while activation of the LTβR pathway has been previously reported in breast, colorectal, lung, larynx/pharynx, gastric cancers [51] and melanomas [52]. Moreover, the nuclear staining for LTβR can be explained by the internalization of LTβR observed upon activation of the alternative NF-κB pathway [53] and the presence of a specific motif on LTβR that is responsible for its subcellular compartmentalization [54]. However, in contrast to BAFFR, no more data exists on the role of LTβR in the nucleus; therefore, further research is needed to elucidate it. 

Another intriguing finding is the association of CD40 expression in TILs and in CAFs with the development of metastatic disease (adrenal glands, brain and liver). Supportive to our finding is the association of CD40 expression with metastatic spread of human lung cancer cells in vitro [25]. Moreover, CD40 protein expression appears to be correlated with lymph node infiltration in lung adenocarcinomas [55], human esophageal squamous cell carcinoma [31], gastric cancer [33] and pancreatic cancer, where it was also associated with the clinical stage of the disease [35]. This finding is further supported by the important role of CD40 demonstrated in the formation of lung metastases from melanoma cells in vivo with mice deficient for CD40 (CD40^−/−^), having a significant reduction in lung metastases after injection with melanoma cells [56,57]. In addition, Brouty-Boyé et al. have shown that human fibroblasts from various tissues express CD40, suggesting a role in immune response [58], while, recently in vitro studies have implicated CAFs in the enhancement of metastatic potential of lung cancer cells [59].

BAFFR tumor or stromal expression was negatively correlated with OS in agreement with transcriptomic data analysis for patients with adenocarcinomas and the well-established knowledge that activation of BAFFR leads to a potent survival signal [60,61]. Additionally, in pancreatic cancer, BAFFR overexpression has been associated with epithelial-mesenchymal transition (EMT) [45], a phenomenon which has an ultimate role in cancer survival [62]. Furthermore, an inverse association of BAFFR expression with clinical outcome (PFS and OS) has also been reported in follicular lymphoma [39] and in diffuse large B-cell lymphoma (DLBCL) patients [63]. These findings are justified by the function of BAFFR as a transcriptional regulator in the nucleus of normal B-cell and B-cell non-Hodgkin lymphoma (NHL-B) cells promoting cell proliferation and survival [61].

A positive association with OS also emerged for *LTβR* gene expression in patients with adenocarcinomas. In addition, LTβR protein expression was related to OS too, with LTβR localization dependency. A negative association with OS was noted for nuclear staining and a positive association was observed for cytoplasmic expression in stage II patients. This finding might reflect a compartment-dependent mechanism for LTβR. Supportive to this is the observation that internalization of LTβR is associated with the activation of the alternative, but not the classical, NF-κB pathway [53]. In addition, it seems that LTβR signaling plays context-dependent roles, exerting either tumor-suppressive or promoting functions in solid tumors [64]. 

Despite our promising results, we have to acknowledge some weak points. In the current study, patients with stage I, II and III NSCLC, who were surgically managed, were exclusively enrolled, limiting the investigation to non-metastatic disease. In addition, a larger cohort would be required to achieve more robust results. Furthermore, although IHC is a widely used technique to assess protein expression, it would be reasonable to have data from multiplex techniques. Moreover, double staining could help in discrimination of the different types of cells, which was based on morphological characteristics.

In conclusion, this study shows that although mRNA expression levels of the immune system-related transmembrane receptors CD40, BAFFR, RANK and LTβR do not differ, protein levels of CD40 and BAFFR were lower in NSCLC while no change was observed in protein levels of RANK and LTβR. Moreover, CD40 and BAFFR protein expression in microenvironment cells were correlated with development of metastasis. In addition, our data provide strong evidence that the expression of these receptors in tumor, immune or stroma cells have prognostic significance influencing the clinical outcome of operated NSCLC patients, while CD40 and BAFFR protein expression was correlated with development of distant metastases.

## Figures and Tables

**Table 1:** Primary antibodies and their clonality, clone, dilution, antigen retrieval and incubation time information. Abbreviations: RANK; Receptor Activator of NF-κB, LTβR; Lymphotoxin β receptor, CD40; Tumor necrosis factor receptor superfamily member 5, BAFFR; B-cell activating factor receptor, M; monoclonal, P; polyclonal.

Antibody	Clonality/Source	Company	Catalogue number	Clone	Dilution	Antigen Retrieval Conditions	Incubation Time
RANK	M/mouse	R&D systems Minneapolis, MN, USA	MAB6831	80707	1:300	1.2 mM EDTA, pH 8.0	Overnight 4 °C
LTβR	P/rabbit	Abcam Cambridge, UK	Ab193449	-	1:750	8 mM sodium citrate, pH 6.0	Overnight 4 °C
CD40	M/mouse	SANTA CRUZ, Biotechnology Dallas, TX, USA	Sc-13528	LOB-11	1:20	1.2 mM EDTA, pH 8.0	Overnight 4 °C
BAFFR	M/mouse	SANTA CRUZ, Biotechnology Dallas, TX, USA	Sc-32774	11C1	1:20	1.2 mM EDTA, pH 8.0	Overnight 4 °C

**Table 2:** Clinicopathological characteristics and survival data of non-small-cell lung cancer (NSCLC) patients. Abbreviations: NA, data not available or unknown.

Clinicopathological Characteristics	Cases *n* (%)
**Total**	**119 (100)**
**Age (years) Median (range)**	**66 (42–84)**
**Gender**	
**Total**	**119 (100)**
**Male**	**112 (94.1)**
**Female**	**7 (5.9)**
**Smoking (pack-years)**	
**Total**	**119 (100)**
**Cases (%)**	**49 (41.2)**
**Mean (range)**	**90.31 (20–165)**
**NA**	**90 (58.8)**
**Primary location**	
**Total**	**119 (100)**
**Left lung**	**45 (37.8)**
**Right lung**	**74 (62.2)**
**NA**	**-**
**Histology**	
**Total**	**119 (100)**
**Squamous**	**67 (56.3)**
**Adenocarcinoma**	**42 (35.3)**
**Large carcinoma**	**10 (8.4)**
**NA**	**-**
**Stage**	
**Total**	**119 (100)**
**I**	**43 (36.1)**
**II**	**37 (31.1)**
**III**	**39 (32.8)**
**NA**	**-**
**Grade**	
**Total**	**119 (100)**
**I**	**5 (4.2)**
**II**	**55 (46.2)**
**III**	**52 (43.7)**
**NA**	**7 (5.9)**
**Maximum diameter (cm)**	
**Total**	**119 (100)**
**Cases (%)**	**108 (99.2)**
**Mean (range)**	**5.35 (1.10–21.00)**
**NA**	**1 (0.8)**
**Lymph node infiltration**	
**Total**	**119 (100)**
**No**	**63 (52.9)**
**Yes**	**52 (43.7)**
**NA**	**4 (3.4)**
**Metastasis (adrenals)**	
**Total**	**119 (100)**
**No**	**24 (20.2)**
**Yes**	**3 (2.5)**
**NA**	**92 (77.3)**
**Metastasis (liver)**	
**Total**	**119 (100)**
**No**	**25 (21.0)**
**Yes**	**4 (3.4)**
**NA**	**90 (75.6)**
**Metastasis (brain)**	
**Total**	**119 (100)**
**No**	**24 (20.2)**
**Yes**	**7 (5.9)**
**NA**	**88 (73.9)**
**Metastasis (bone)**	
**Total**	**119 (100)**
**No**	**19 (16.0)**
**Yes**	**14 (11.8)**
**NA**	**86 (72.3)**
**Metastasis (adrenals-liver-brain-bone)**	
**Total**	**119 (100)**
**No**	**8 (6.7)**
**Yes**	**28 (23.5)**
**NA**	**83 (69.7)**
**Survival (two years)**	
**Total**	**119 (100)**
**Dead**	**46 (38.7)**
**Alive**	**71 (59.7)**
**NA**	**2 (1.7)**
**Survival (three years)**	
**Total**	**119 (100)**
**Dead**	**59 (49.6)**
**Alive**	**56 (47.1)**
**NA**	**4 (3.4)**
**Survival (five years)**	
**Total**	**119 (100)**
**Dead**	**70 (58.8)**
**Alive**	**45 (37.8)**
**NA**	**4 (3.4)**
**Relapse**	
**Total**	**119 (100)**
**No**	**14 (11.8)**
**Yes**	**16 (13.4)**
**NA**	**89 (74.8)**
